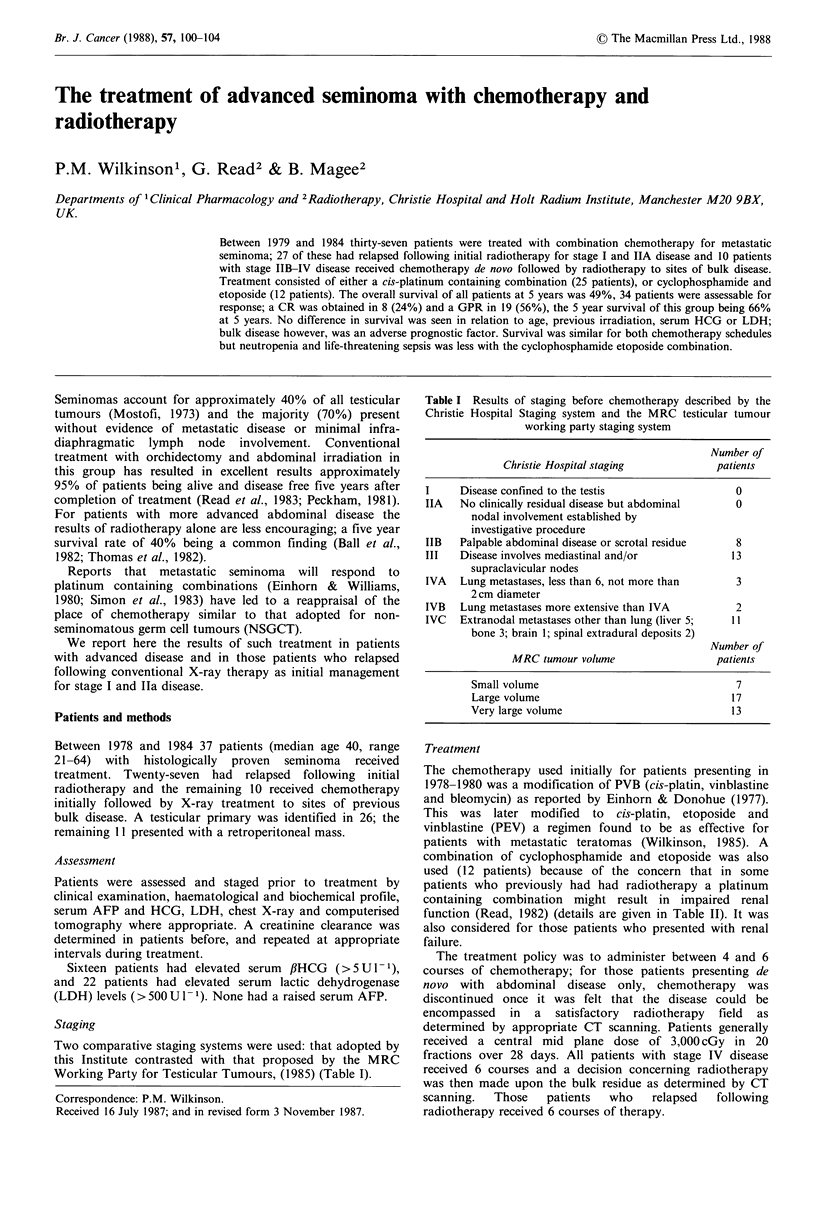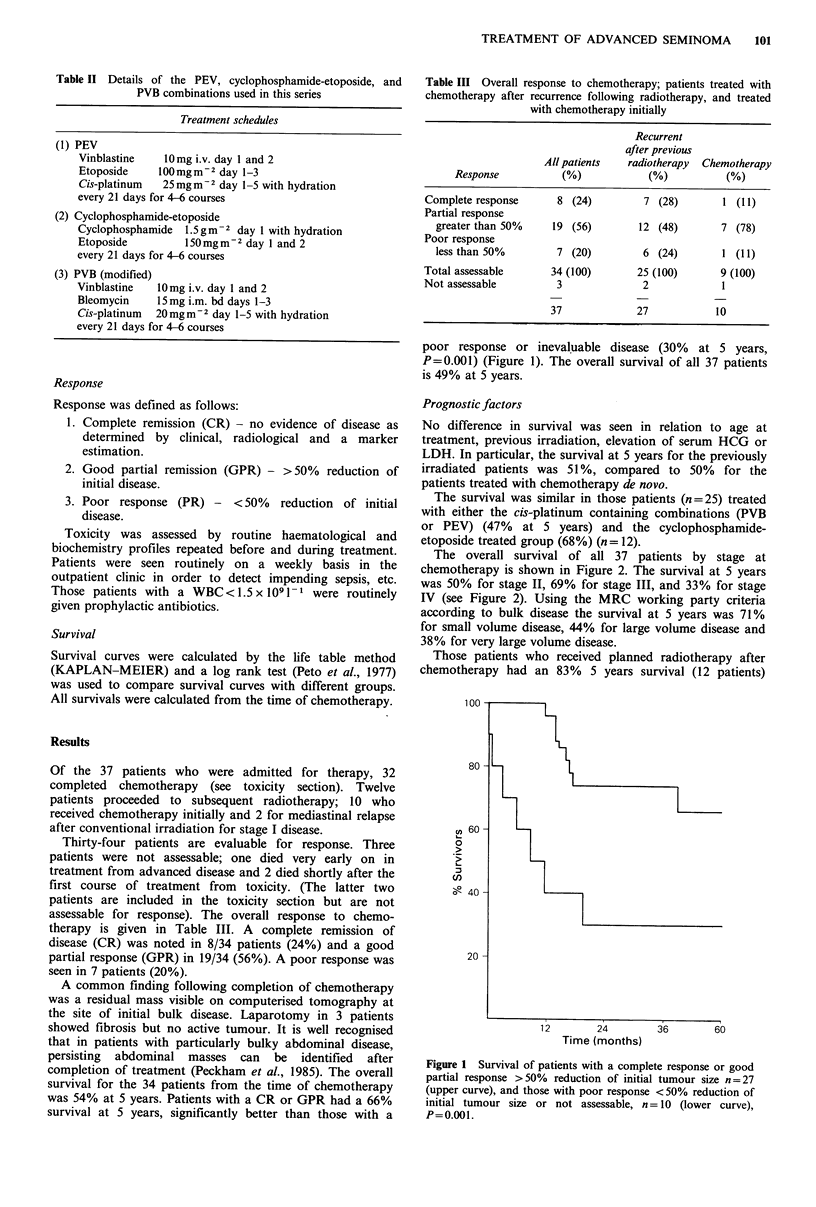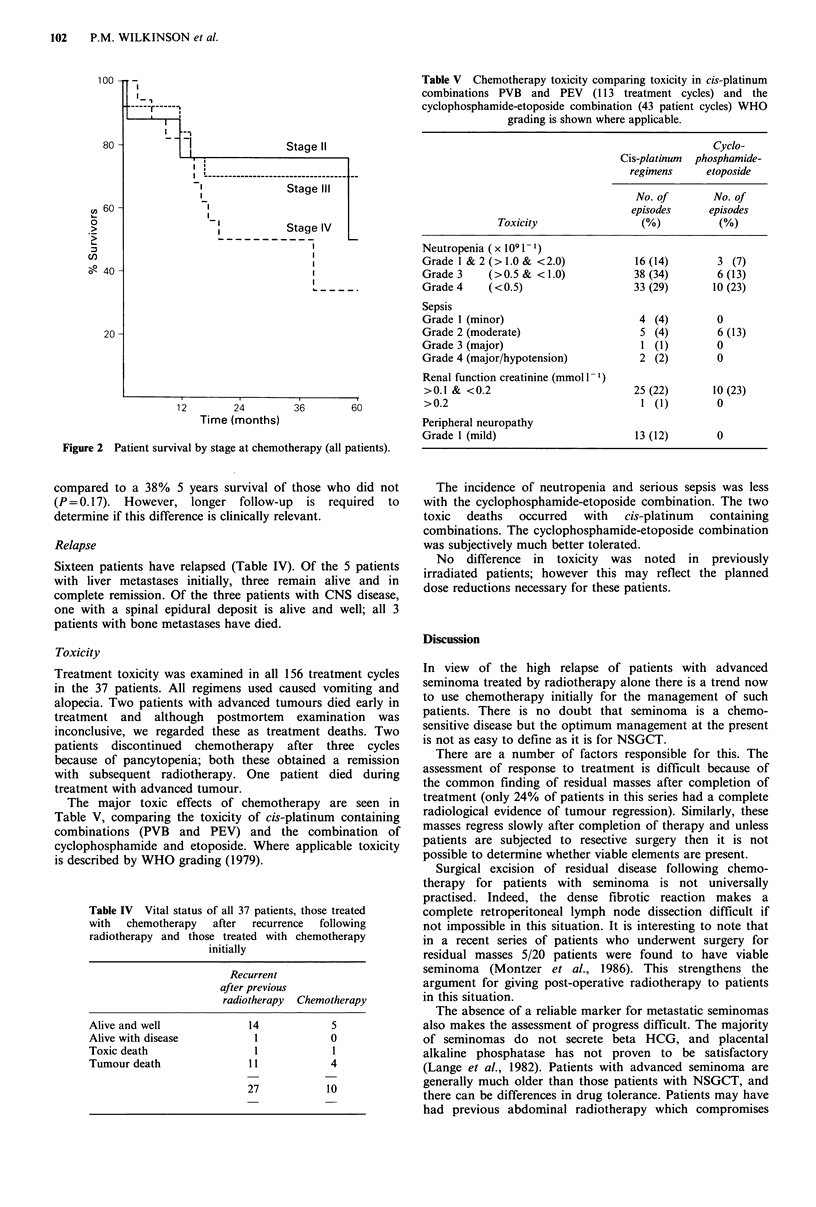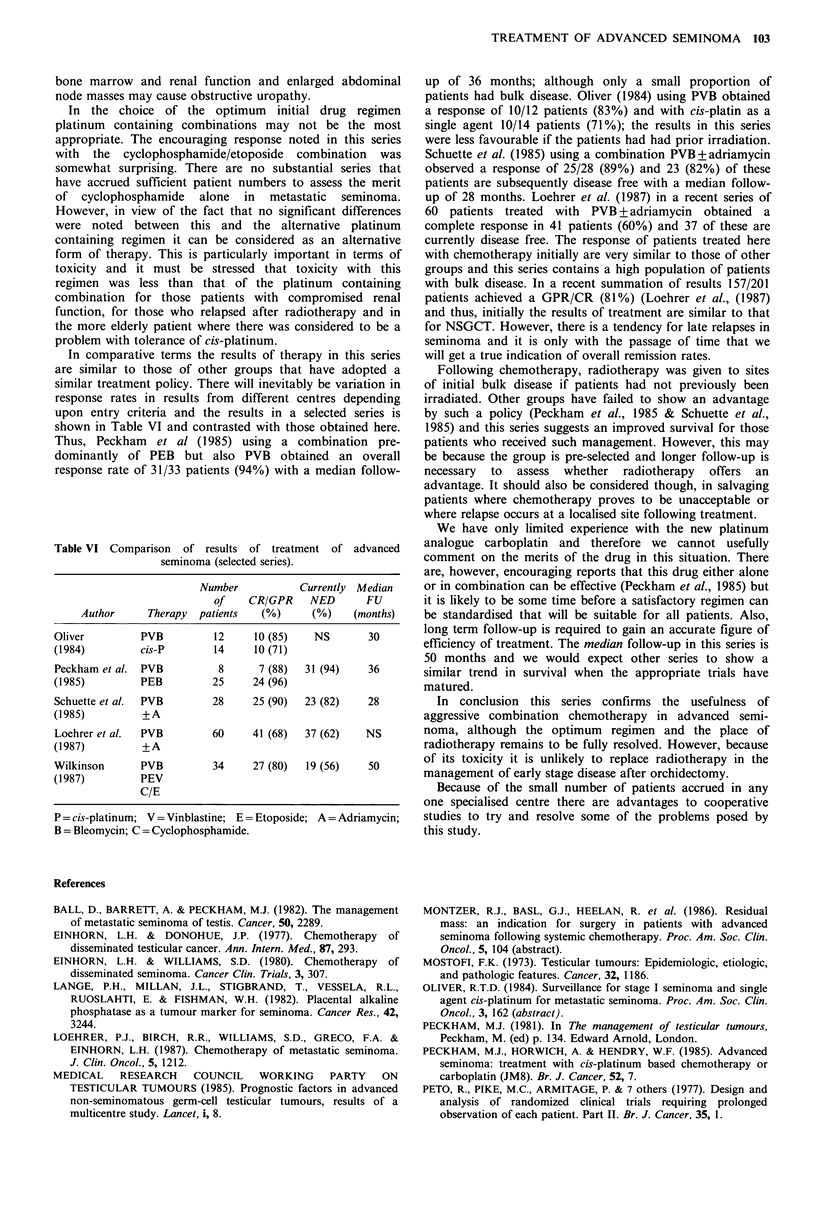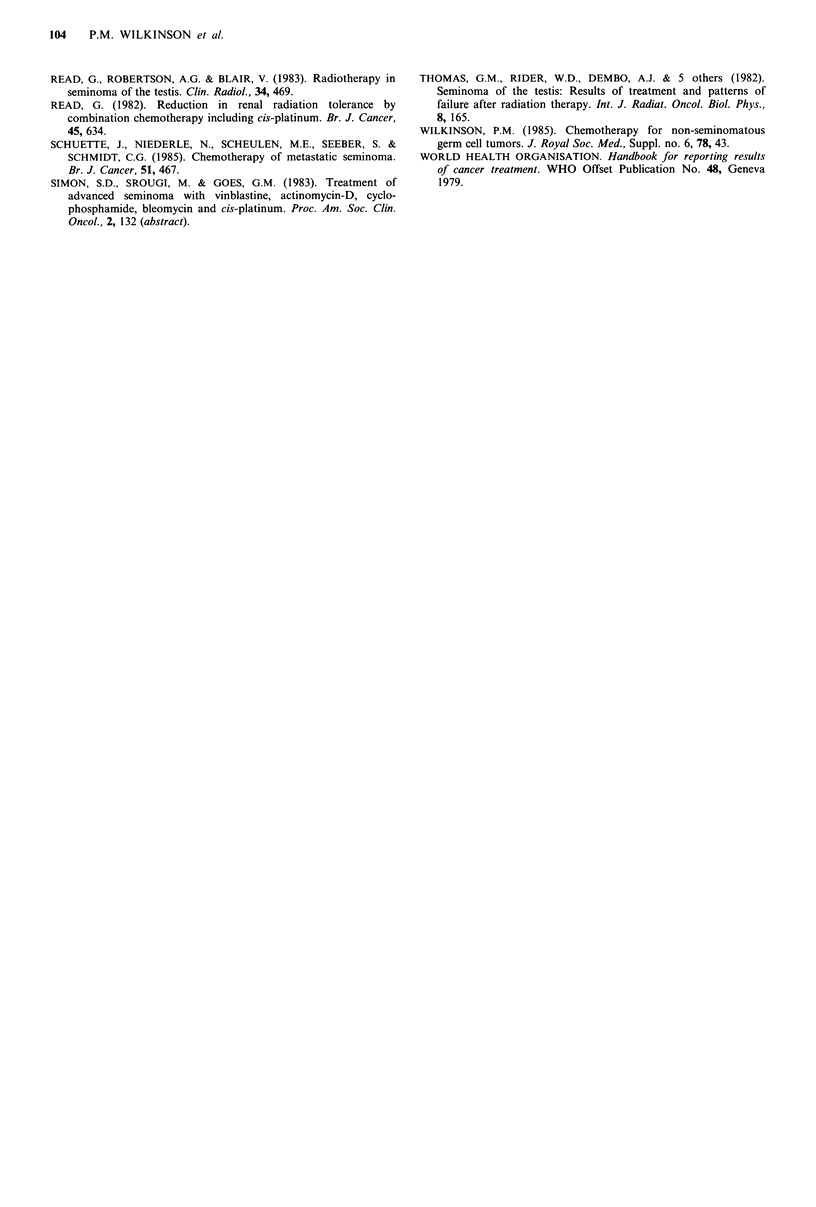# The treatment of advanced seminoma with chemotherapy and radiotherapy.

**DOI:** 10.1038/bjc.1988.18

**Published:** 1988-01

**Authors:** P. M. Wilkinson, G. Read, B. Magee

**Affiliations:** Department of Clinical Pharmacology, Christie Hospital and Holt Radium Institute, Manchester, UK.

## Abstract

Between 1979 and 1984 thirty-seven patients were treated with combination chemotherapy for metastatic seminoma; 27 of these had relapsed following initial radiotherapy for stage I and IIA disease and 10 patients with stage IIB-IV disease received chemotherapy de novo followed by radiotherapy to sites of bulk disease. Treatment consisted of either a cis-platinum containing combination (25 patients), or cyclophosphamide and etoposide (12 patients). The overall survival of all patients at 5 years was 49%, 34 patients were assessable for response; a CR was obtained in 8 (24%) and a GPR in 19 (56%), the 5 year survival of this group being 66% at 5 years. No difference in survival was seen in relation to age, previous irradiation, serum HCG or LDH; bulk disease however, was an adverse prognostic factor. Survival was similar for both chemotherapy schedules but neutropenia and life-threatening sepsis was less with the cyclophosphamide etoposide combination.


Br. J. Cancer (1988), 57, 100–104 © The Macmillan Press Ltd., 1988

### The treatment of advanced seminoma with chemotherapy and radiotherapy

P.M. Wilkinson[1], G. Read[2] & B. Magee[2]

*Departments of [1]Clinical Pharmacology and [2]Radiotherapy, Christie Hospital and Holt Radium Institute, Manchester M20 9BX, UK.*

Between 1979 and 1984 thirty-seven patients were treated with combination chemotherapy for metastatic seminoma; 27 of these had relapsed following initial radiotherapy for stage I and IIA disease and 10 patients with stage IIB–IV disease received chemotherapy *de novo* followed by radiotherapy to sites of bulk disease. Treatment consisted of either a *cis*-platinum containing combination (25 patients), or cyclophosphamide and etoposide (12 patients). The overall survival of all patients at 5 years was 49%, 34 patients were assessable for response; a CR was obtained in 8 (24%) and a GPR in 19 (56%), the 5 year survival of this group being 66% at 5 years. No difference in survival was seen in relation to age, previous irradiation, serum HCG or LDH; bulk disease however, was an adverse prognostic factor. Survival was similar for both chemotherapy schedules but neutropenia and life-threatening sepsis was less with the cyclophosphamide etoposide combination.

Seminomas account for approximately 40% of all testicular tumours (Mostofi, 1973) and the majority (70%) present without evidence of metastatic disease or minimal infra-diaphragmatic lymph node involvement. Conventional treatment with orchidectomy and abdominal irradiation in this group has resulted in excellent results approximately 95% of patients being alive and disease free five years after completion of treatment (Read *et al.*, 1983; Peckham, 1981). For patients with more advanced abdominal disease the results of radiotherapy alone are less encouraging; a five year survival rate of 40% being a common finding (Ball *et al.*, 1982; Thomas *et al.*, 1982).

Reports that metastatic seminoma will respond to platinum containing combinations (Einhorn & Williams, 1980; Simon *et al.*, 1983) have led to a reappraisal of the place of chemotherapy similar to that adopted for non-seminomatous germ cell tumours (NSGCT).

We report here the results of such treatment in patients with advanced disease and in those patients who relapsed following conventional X-ray therapy as initial management for stage I and IIa disease.

### Patients and methods

Between 1978 and 1984 37 patients (median age 40, range 21–64) with histologically proven seminoma received treatment. Twenty-seven had relapsed following initial radiotherapy and the remaining 10 received chemotherapy initially followed by X-ray treatment to sites of previous bulk disease. A testicular primary was identified in 26; the remaining 11 presented with a retroperitoneal mass.

### *Assessment*

Patients were assessed and staged prior to treatment by clinical examination, haematological and biochemical profile, serum AFP and HCG, LDH, chest X-ray and computerised tomography where appropriate. A creatinine clearance was determined in patients before, and repeated at appropriate intervals during treatment.

Sixteen patients had elevated serum $\beta$HCG ($>5\,U\,l^{-1}$), and 22 patients had elevated serum lactic dehydrogenase (LDH) levels ($>500\,U\,l^{-1}$). None had a raised serum AFP.

### *Staging*

Two comparative staging systems were used: that adopted by this Institute contrasted with that proposed by the MRC Working Party for Testicular Tumours, (1985) (Table I).

Correspondence: P.M. Wilkinson.
Received 16 July 1987; and in revised form 3 November 1987.

**Table I** Results of staging before chemotherapy described by the Christie Hospital Staging system and the MRC testicular tumour working party staging system

| | *Christie Hospital staging* | *Number of patients* |
|---|---|---|
| I | Disease confined to the testis | 0 |
| IIA | No clinically residual disease but abdominal nodal involvement established by investigative procedure | 0 |
| IIB | Palpable abdominal disease or scrotal residue | 8 |
| III | Disease involves mediastinal and/or supraclavicular nodes | 13 |
| IVA | Lung metastases, less than 6, not more than 2 cm diameter | 3 |
| IVB | Lung metastases more extensive than IVA | 2 |
| IVC | Extranodal metastases other than lung (liver 5; bone 3; brain 1; spinal extradural deposits 2) | 11 |
| | *MRC tumour volume* | *Number of patients* |
| | Small volume | 7 |
| | Large volume | 17 |
| | Very large volume | 13 |

### *Treatment*

The chemotherapy used initially for patients presenting in 1978–1980 was a modification of PVB (*cis*-platin, vinblastine and bleomycin) as reported by Einhorn & Donohue (1977). This was later modified to *cis*-platin, etoposide and vinblastine (PEV) a regimen found to be as effective for patients with metastatic teratomas (Wilkinson, 1985). A combination of cyclophosphamide and etoposide was also used (12 patients) because of the concern that in some patients who previously had had radiotherapy a platinum containing combination might result in impaired renal function (Read, 1982) (details are given in Table II). It was also considered for those patients who presented with renal failure.

The treatment policy was to administer between 4 and 6 courses of chemotherapy; for those patients presenting *de novo* with abdominal disease only, chemotherapy was discontinued once it was felt that the disease could be encompassed in a satisfactory radiotherapy field as determined by appropriate CT scanning. Patients generally received a central mid plane dose of 3,000 cGy in 20 fractions over 28 days. All patients with stage IV disease received 6 courses and a decision concerning radiotherapy was then made upon the bulk residue as determined by CT scanning. Those patients who relapsed following radiotherapy received 6 courses of therapy.

**Table II** Details of the PEV, cyclophosphamide-etoposide, and PVB combinations used in this series

| *Treatment schedules* | |
|---|---|
| (1) PEV | |
| Vinblastine | 10 mg i.v. day 1 and 2 |
| Etoposide | 100 mg m$^{-2}$ day 1–3 |
| *Cis*-platinum | 25 mg m$^{-2}$ day 1–5 with hydration |
| every 21 days for 4–6 courses | |
| (2) Cyclophosphamide-etoposide | |
| Cyclophosphamide | 1.5 g m$^{-2}$ day 1 with hydration |
| Etoposide | 150 mg m$^{-2}$ day 1 and 2 |
| every 21 days for 4–6 courses | |
| (3) PVB (modified) | |
| Vinblastine | 10 mg i.v. day 1 and 2 |
| Bleomycin | 15 mg i.m. bd days 1–3 |
| *Cis*-platinum | 20 mg m$^{-2}$ day 1–5 with hydration |
| every 21 days for 4–6 courses | |

*Response*

Response was defined as follows:

1. Complete remission (CR) – no evidence of disease as determined by clinical, radiological and a marker estimation.
2. Good partial remission (GPR) – >50% reduction of initial disease.
3. Poor response (PR) – <50% reduction of initial disease.

Toxicity was assessed by routine haematological and biochemistry profiles repeated before and during treatment. Patients were seen routinely on a weekly basis in the outpatient clinic in order to detect impending sepsis, etc. Those patients with a WBC $<1.5\times10^{9}\,l^{-1}$ were routinely given prophylactic antibiotics.

*Survival*

Survival curves were calculated by the life table method (KAPLAN–MEIER) and a log rank test (Peto *et al.*, 1977) was used to compare survival curves with different groups. All survivals were calculated from the time of chemotherapy.

### Results

Of the 37 patients who were admitted for therapy, 32 completed chemotherapy (see toxicity section). Twelve patients proceeded to subsequent radiotherapy; 10 who received chemotherapy initially and 2 for mediastinal relapse after conventional irradiation for stage I disease.

Thirty-four patients are evaluable for response. Three patients were not assessable; one died very early on in treatment from advanced disease and 2 died shortly after the first course of treatment from toxicity. (The latter two patients are included in the toxicity section but are not assessable for response). The overall response to chemotherapy is given in Table III. A complete remission of disease (CR) was noted in 8/34 patients (24%) and a good partial response (GPR) in 19/34 (56%). A poor response was seen in 7 patients (20%).

A common finding following completion of chemotherapy was a residual mass visible on computerised tomography at the site of initial bulk disease. Laparotomy in 3 patients showed fibrosis but no active tumour. It is well recognised that in patients with particularly bulky abdominal disease, persisting abdominal masses can be identified after completion of treatment (Peckham *et al.*, 1985). The overall survival for the 34 patients from the time of chemotherapy was 54% at 5 years. Patients with a CR or GPR had a 66% survival at 5 years, significantly better than those with a

**Table III** Overall response to chemotherapy; patients treated with chemotherapy after recurrence following radiotherapy, and treated with chemotherapy initially

| *Response* | *All patients* (%) | *Recurrent after previous radiotherapy* (%) | *Chemotherapy* (%) |
|---|---|---|---|
| Complete response | 8 (24) | 7 (28) | 1 (11) |
| Partial response greater than 50% | 19 (56) | 12 (48) | 7 (78) |
| Poor response less than 50% | 7 (20) | 6 (24) | 1 (11) |
| Total assessable | 34 (100) | 25 (100) | 9 (100) |
| Not assessable | 3 | 2 | 1 |
| | 37 | 27 | 10 |

poor response or inevaluable disease (30% at 5 years, $P=0.001$) (Figure 1). The overall survival of all 37 patients is 49% at 5 years.

*Prognostic factors*

No difference in survival was seen in relation to age at treatment, previous irradiation, elevation of serum HCG or LDH. In particular, the survival at 5 years for the previously irradiated patients was 51%, compared to 50% for the patients treated with chemotherapy *de novo*.

The survival was similar in those patients ($n=25$) treated with either the *cis*-platinum containing combinations (PVB or PEV) (47% at 5 years) and the cyclophosphamide-etoposide treated group (68%) ($n=12$).

The overall survival of all 37 patients by stage at chemotherapy is shown in Figure 2. The survival at 5 years was 50% for stage II, 69% for stage III, and 33% for stage IV (see Figure 2). Using the MRC working party criteria according to bulk disease the survival at 5 years was 71% for small volume disease, 44% for large volume disease and 38% for very large volume disease.

Those patients who received planned radiotherapy after chemotherapy had an 83% 5 years survival (12 patients)

**Figure 1** Survival of patients with a complete response or good partial response >50% reduction of initial tumour size $n=27$ (upper curve), and those with poor response <50% reduction of initial tumour size or not assessable, $n=10$ (lower curve), $P=0.001$.

**Figure 2** Patient survival by stage at chemotherapy (all patients).

compared to a 38% 5 years survival of those who did not ($P=0.17$). However, longer follow-up is required to determine if this difference is clinically relevant.

*Relapse*

Sixteen patients have relapsed (Table IV). Of the 5 patients with liver metastases initially, three remain alive and in complete remission. Of the three patients with CNS disease, one with a spinal epidural deposit is alive and well; all 3 patients with bone metastases have died.

*Toxicity*

Treatment toxicity was examined in all 156 treatment cycles in the 37 patients. All regimens used caused vomiting and alopecia. Two patients with advanced tumours died early in treatment and although postmortem examination was inconclusive, we regarded these as treatment deaths. Two patients discontinued chemotherapy after three cycles because of pancytopenia; both these obtained a remission with subsequent radiotherapy. One patient died during treatment with advanced tumour.

The major toxic effects of chemotherapy are seen in Table V, comparing the toxicity of *cis*-platinum containing combinations (PVB and PEV) and the combination of cyclophosphamide and etoposide. Where applicable toxicity is described by WHO grading (1979).

**Table IV** Vital status of all 37 patients, those treated with chemotherapy after recurrence following radiotherapy and those treated with chemotherapy initially

| | *Recurrent after previous radiotherapy* | *Chemotherapy* |
|---|---|---|
| Alive and well | 14 | 5 |
| Alive with disease | 1 | 0 |
| Toxic death | 1 | 1 |
| Tumour death | 11 | 4 |
| | 27 | 10 |

**Table V** Chemotherapy toxicity comparing toxicity in *cis*-platinum combinations PVB and PEV (113 treatment cycles) and the cyclophosphamide-etoposide combination (43 patient cycles) WHO grading is shown where applicable.

| | Cis-*platinum regimens* | *Cyclo-phosphamide-etoposide* |
|---|---|---|
| *Toxicity* | *No. of episodes* (%) | *No. of episodes* (%) |
| Neutropenia ($\times 10^9 l^{-1}$) | | |
| Grade 1 & 2 (>1.0 & <2.0) | 16 (14) | 3 (7) |
| Grade 3 (>0.5 & <1.0) | 38 (34) | 6 (13) |
| Grade 4 (<0.5) | 33 (29) | 10 (23) |
| Sepsis | | |
| Grade 1 (minor) | 4 (4) | 0 |
| Grade 2 (moderate) | 5 (4) | 6 (13) |
| Grade 3 (major) | 1 (1) | 0 |
| Grade 4 (major/hypotension) | 2 (2) | 0 |
| Renal function creatinine ($mmol\,l^{-1}$) | | |
| >0.1 & <0.2 | 25 (22) | 10 (23) |
| >0.2 | 1 (1) | 0 |
| Peripheral neuropathy | | |
| Grade 1 (mild) | 13 (12) | 0 |

The incidence of neutropenia and serious sepsis was less with the cyclophosphamide-etoposide combination. The two toxic deaths occurred with *cis*-platinum containing combinations. The cyclophosphamide-etoposide combination was subjectively much better tolerated.

No difference in toxicity was noted in previously irradiated patients; however this may reflect the planned dose reductions necessary for these patients.

### Discussion

In view of the high relapse of patients with advanced seminoma treated by radiotherapy alone there is a trend now to use chemotherapy initially for the management of such patients. There is no doubt that seminoma is a chemo-sensitive disease but the optimum management at the present is not as easy to define as it is for NSGCT.

There are a number of factors responsible for this. The assessment of response to treatment is difficult because of the common finding of residual masses after completion of treatment (only 24% of patients in this series had a complete radiological evidence of tumour regression). Similarly, these masses regress slowly after completion of therapy and unless patients are subjected to resective surgery then it is not possible to determine whether viable elements are present.

Surgical excision of residual disease following chemotherapy for patients with seminoma is not universally practised. Indeed, the dense fibrotic reaction makes a complete retroperitoneal lymph node dissection difficult if not impossible in this situation. It is interesting to note that in a recent series of patients who underwent surgery for residual masses 5/20 patients were found to have viable seminoma (Montzer *et al.*, 1986). This strengthens the argument for giving post-operative radiotherapy to patients in this situation.

The absence of a reliable marker for metastatic seminomas also makes the assessment of progress difficult. The majority of seminomas do not secrete beta HCG, and placental alkaline phosphatase has not proven to be satisfactory (Lange *et al.*, 1982). Patients with advanced seminoma are generally much older than those patients with NSGCT, and there can be differences in drug tolerance. Patients may have had previous abdominal radiotherapy which compromises bone marrow and renal function and enlarged abdominal node masses may cause obstructive uropathy.

In the choice of the optimum initial drug regimen platinum containing combinations may not be the most appropriate. The encouraging response noted in this series with the cyclophosphamide/etoposide combination was somewhat surprising. There are no substantial series that have accrued sufficient patient numbers to assess the merit of cyclophosphamide alone in metastatic seminoma. However, in view of the fact that no significant differences were noted between this and the alternative platinum containing regimen it can be considered as an alternative form of therapy. This is particularly important in terms of toxicity and it must be stressed that toxicity with this regimen was less than that of the platinum containing combination for those patients with compromised renal function, for those who relapsed after radiotherapy and in the more elderly patient where there was considered to be a problem with tolerance of *cis*-platinum.

In comparative terms the results of therapy in this series are similar to those of other groups that have adopted a similar treatment policy. There will inevitably be variation in response rates in results from different centres depending upon entry criteria and the results in a selected series is shown in Table VI and contrasted with those obtained here. Thus, Peckham *et al* (1985) using a combination predominantly of PEB but also PVB obtained an overall response rate of 31/33 patients (94%) with a median follow-up of 36 months; although only a small proportion of patients had bulk disease. Oliver (1984) using PVB obtained a response of 10/12 patients (83%) and with *cis*-platin as a single agent 10/14 patients (71%); the results in this series were less favourable if the patients had had prior irradiation. Schuette *et al.* (1985) using a combination PVB ± adriamycin observed a response of 25/28 (89%) and 23 (82%) of these patients are subsequently disease free with a median follow-up of 28 months. Loehrer *et al.* (1987) in a recent series of 60 patients treated with PVB ± adriamycin obtained a complete response in 41 patients (60%) and 37 of these are currently disease free. The response of patients treated here with chemotherapy initially are very similar to those of other groups and this series contains a high population of patients with bulk disease. In a recent summation of results 157/201 patients achieved a GPR/CR (81%) (Loehrer *et al.*, (1987) and thus, initially the results of treatment are similar to that for NSGCT. However, there is a tendency for late relapses in seminoma and it is only with the passage of time that we will get a true indication of overall remission rates.

**Table VI** Comparison of results of treatment of advanced seminoma (selected series).

| *Author* | *Therapy* | *Number of patients* | *CR/GPR (%)* | *Currently NED (%)* | *Median FU (months)* |
|---|---|---|---|---|---|
| Oliver (1984) | PVB<br>*cis*-P | 12<br>14 | 10 (85)<br>10 (71) | NS | 30 |
| Peckham *et al.* (1985) | PVB<br>PEB | 8<br>25 | 7 (88)<br>24 (96) | 31 (94) | 36 |
| Schuette *et al.* (1985) | PVB<br>±A | 28 | 25 (90) | 23 (82) | 28 |
| Loehrer *et al.* (1987) | PVB<br>±A | 60 | 41 (68) | 37 (62) | NS |
| Wilkinson (1987) | PVB<br>PEV<br>C/E | 34 | 27 (80) | 19 (56) | 50 |

P = *cis*-platinum; V = Vinblastine; E = Etoposide; A = Adriamycin; B = Bleomycin; C = Cyclophosphamide.

Following chemotherapy, radiotherapy was given to sites of initial bulk disease if patients had not previously been irradiated. Other groups have failed to show an advantage by such a policy (Peckham *et al.*, 1985 & Schuette *et al.*, 1985) and this series suggests an improved survival for those patients who received such management. However, this may be because the group is pre-selected and longer follow-up is necessary to assess whether radiotherapy offers an advantage. It should also be considered though, in salvaging patients where chemotherapy proves to be unacceptable or where relapse occurs at a localised site following treatment.

We have only limited experience with the new platinum analogue carboplatin and therefore we cannot usefully comment on the merits of the drug in this situation. There are, however, encouraging reports that this drug either alone or in combination can be effective (Peckham *et al.*, 1985) but it is likely to be some time before a satisfactory regimen can be standardised that will be suitable for all patients. Also, long term follow-up is required to gain an accurate figure of efficiency of treatment. The *median* follow-up in this series is 50 months and we would expect other series to show a similar trend in survival when the appropriate trials have matured.

In conclusion this series confirms the usefulness of aggressive combination chemotherapy in advanced seminoma, although the optimum regimen and the place of radiotherapy remains to be fully resolved. However, because of its toxicity it is unlikely to replace radiotherapy in the management of early stage disease after orchidectomy.

Because of the small number of patients accrued in any one specialised centre there are advantages to cooperative studies to try and resolve some of the problems posed by this study.

### References

BALL, D., BARRETT, A. & PECKHAM, M.J. (1982). The management of metastatic seminoma of testis. *Cancer*, **50**, 2289.

EINHORN, L.H. & DONOHUE, J.P. (1977). Chemotherapy of disseminated testicular cancer. *Ann. Intern. Med.*, **87**, 293.

EINHORN, L.H. & WILLIAMS, S.D. (1980). Chemotherapy of disseminated seminoma. *Cancer Clin. Trials*, **3**, 307.

LANGE, P.H., MILLAN, J.L., STIGBRAND, T., VESSELA, R.L., RUOSLAHTI, E. & FISHMAN, W.H. (1982). Placental alkaline phosphatase as a tumour marker for seminoma. *Cancer Res.*, **42**, 3244.

LOEHRER, P.J., BIRCH, R.R., WILLIAMS, S.D., GRECO, F.A. & EINHORN, L.H. (1987). Chemotherapy of metastatic seminoma. *J. Clin. Oncol.*, **5**, 1212.

MEDICAL RESEARCH COUNCIL WORKING PARTY ON TESTICULAR TUMOURS (1985). Prognostic factors in advanced non-seminomatous germ-cell testicular tumours, results of a multicentre study. *Lancet*, **i**, 8.

MONTZER, R.J., BASL, G.J., HEELAN, R. *et al.* (1986). Residual mass: an indication for surgery in patients with advanced seminoma following systemic chemotherapy. *Proc. Am. Soc. Clin. Oncol.*, **5**, 104 (abstract).

MOSTOFI, F.K. (1973). Testicular tumours: Epidemiologic, etiologic, and pathologic features. *Cancer*, **32**, 1186.

OLIVER, R.T.D. (1984). Surveillance for stage I seminoma and single agent *cis*-platinum for metastatic seminoma. *Proc. Am. Soc. Clin. Oncol.*, **3**, 162 (*abstract*).

PECKHAM, M.J. (1981). In *The management of testicular tumours*, Peckham, M. (ed) p. 134. Edward Arnold, London.

PECKHAM, M.J., HORWICH, A. & HENDRY, W.F. (1985). Advanced seminoma: treatment with *cis*-platinum based chemotherapy or carboplatin (JM8). *Br. J. Cancer*, **52**, 7.

PETO, R., PIKE, M.C., ARMITAGE, P. & 7 others (1977). Design and analysis of randomized clinical trials requiring prolonged observation of each patient. Part II. *Br. J. Cancer*, **35**, 1.

READ, G., ROBERTSON, A.G. & BLAIR, V. (1983). Radiotherapy in seminoma of the testis. *Clin. Radiol.*, **34**, 469.

READ, G. (1982). Reduction in renal radiation tolerance by combination chemotherapy including *cis*-platinum. *Br. J. Cancer*, **45**, 634.

SCHUETTE, J., NIEDERLE, N., SCHEULEN, M.E., SEEBER, S. & SCHMIDT, C.G. (1985). Chemotherapy of metastatic seminoma. *Br. J. Cancer*, **51**, 467.

SIMON, S.D., SROUGI, M. & GOES, G.M. (1983). Treatment of advanced seminoma with vinblastine, actinomycin-D, cyclophosphamide, bleomycin and *cis*-platinum. *Proc. Am. Soc. Clin. Oncol.*, **2**, 132 (*abstract*).

THOMAS, G.M., RIDER, W.D., DEMBO, A.J. & 5 others (1982). Seminoma of the testis: Results of treatment and patterns of failure after radiation therapy. *Int. J. Radiat. Oncol. Biol. Phys.*, **8**, 165.

WILKINSON, P.M. (1985). Chemotherapy for non-seminomatous germ cell tumors. *J. Royal Soc. Med.*, Suppl. no. 6, **78**, 43.

WORLD HEALTH ORGANISATION. *Handbook for reporting results of cancer treatment.* WHO Offset Publication No. **48**, Geneva 1979.